# A Biohybrid Material With Extracellular Matrix Core and Polymeric Coating as a Cell Honing Cardiovascular Tissue Substitute

**DOI:** 10.3389/fcvm.2022.807255

**Published:** 2022-03-24

**Authors:** Jahnavi Mudigonda, Dongyang Xu, Alan Amedi, Brooks A. Lane, Daniella Corporan, Vivian Wang, Muralidhar Padala

**Affiliations:** ^1^Structural Heart Research & Innovation Laboratory, Carlyle Fraser Heart Center, Emory University Hospital Midtown, Atlanta, GA, United States; ^2^Division of Cardiothoracic Surgery, Emory University School of Medicine, Atlanta, GA, United States

**Keywords:** biomaterial, tissue engineering, xenogeneic, cardiovascular, extracellular matrix

## Abstract

**Objective:**

To investigate the feasibility of a hybrid material in which decellularized pericardial extracellular matrix is functionalized with polymeric nanofibers, for use as a cardiovascular tissue substitute.

**Background:**

A cardiovascular tissue substitute, which is gradually resorbed and is replaced by host's native tissue, has several advantages. Especially in children and young adults, a resorbable material can be useful in accommodating growth, but also enable rapid endothelialization that is necessary to avoid thrombotic complications. In this study, we report a hybrid material, wherein decellularized pericardial matrix is functionalized with a layer of polymeric nanofibers, to achieve the mechanical strength for implantation in the cardiovascular system, but also have enhanced cell honing capacity.

**Methods:**

Pericardial sacs were decellularized with sodium deoxycholate, and polycaprolactone-chitosan fibers were electrospun onto the matrix. Tissue-polymer interaction was evaluated using spectroscopic methods, and the mechanical properties of the individual components and the hybrid material were quantified. *In-vitro* blood flow loop studies were conducted to assess hemocompatibility and cell culture methods were used to assess biocompatibility.

**Results:**

Encapsulation of the decellularized matrix with 70 μm thick matrix of polycaprolactone-chitosan nanofibers, was feasible and reproducible. Spectroscopy of the cross-section depicted new amide bond formation and C–O–C stretch at the interface. An average peel strength of 56.13 ± 11.87 mN/mm^2^ was measured, that is sufficient to withstand a high shear of 15 dynes/cm^2^ without delamination. Mechanical strength and extensibility ratio of the decellularized matrix alone were 18,000 ± 4,200 KPa and 0.18 ± 0.03% whereas that of the hybrid was higher at 20,000 ± 6,600 KPa and 0.35 ± 0.20%. Anisotropy index and stiffness of the biohybrid were increased as well. Neither thrombus formation, nor platelet adhesion or hemolysis was measured in the *in-vitro* blood flow loop studies. Cellular adhesion and survival were adequate in the material.

**Conclusion:**

Encapsulating a decellularized matrix with a polymeric nanofiber coating, has favorable attributes for use as a cardiovascular tissue substitute.

## Introduction

Cardiovascular surgical procedures often require tissue reconstruction, and appropriate materials for this purpose are lacking. Autologous tissue from the patient would be the most ideal, but such materials are scarcely available and are variable in quality. Thus, non-autologous or fully synthetic materials are needed to fulfill this gap, with the most ideal material being one that gradually resorbs and integrates into the host's body (1). This is especially desirable in children undergoing cardiac surgery where long-term durability, hemocompatibility, and implant remodeling are essential to withstand pulsatile hemodynamics and somatic growth.

There is currently a dearth of such materials and this gap is acknowledged by the scientific community (2–5). Currently, glutaraldehyde-fixed bovine pericardium (Glut BP), that was introduced in the 1970s is utilized for its hemocompatibility and mechanical strength. This material is used widely, both as surgical patches and as leaflets in prosthetic heart valves (6). Glutaraldehyde, a common fixative, makes the BP inert to bioactivity and hydrophobic and increases its mechanical strength by crosslinking collagen fibers in the BP, making it suitable for use in the cardiovascular system. However, it also can promote tissue calcification and lead to structural deterioration of the material over time (7, 8). Glycation and albumin infiltration into these materials has also been shown to occur, leading to non-calcific tissue deterioration as well (9). These shortcomings make it less than ideal for long-term efficacy and host integration (1, 4, 6, 10, 11).

To overcome these challenges, glutaraldehyde-free, detergent-based decellularized pericardia have been introduced. Though of superior immunogenicity, these tissues lack adequate mechanical strength for use in the cardiovascular system (12). Cell seeding and *in-vitro* mechanical preconditioning were tried, but such attempts have also not yielded tangible outcomes that could improve their clinical translation (13, 14). The failure mechanisms are often attributed to weak mechanical properties, residual detergents, and remnant cellular materials that cause structural degeneration and calcification and prevents active remodeling (15–17). Synthetic grafts made from polymers, such as Gore-Tex and Dacron, have gained significant use in the past few decades due to excellent mechanical properties, and some success in achieving hemocompatibility (2, 3, 18). However, the immune response elicited by these grafts creates fibrosis and calcification, reproducing the challenges observed with currently used materials (19).

Recently, a distinct *in-situ* tissue engineering approach has gained traction, in which synthetic materials that are inert at the time of implantation, but remodel and are resorbed *in situ* are being used. These are made from biodegradable supramolecular polymers, which elicit a host immune response, but gradually breakdown and are replaced by the own tissue of the host (12, 20, 21). Successful remodeling of these materials is dependent on the chemical nature of the material, its porosity and surface profile that enables cellular attachment, degradation profile in relation to the host tissue formation, and hemocompatibility. Tissue engineered materials so far have been very promising in animal models with short and intermediate follow-ups (22–25) and some of these materials are being tested preclinically with some success (24, 26). Though the preclinical results are promising, the long-term immune response and mechanistic studies that elucidate long-term fibrotic phenotypes are unknown (1). One of the main challenges for designing a desired acellular biomaterial is to achieve a balance between scaffold degradation and neotissue formation without eliciting unfavorable chronic immune and fibrotic response (15, 22, 27, 28).

Hybrid tissue engineering is a more recent approach, in which instead of using a fully synthetic degradable scaffold, a combination of two materials is used. Often, one of the materials is a synthetic polymer that baits cells from the host, in a programmed manner and the second material is another natural polymer or a native decellularized matrix that provides a 3-dimensional scaffold for the cells to hone into and thrive (29–35). Combining synthetic and natural polymers allows programmed mechanical properties, but is susceptible to enzymatic degradation over time (6, 30). On the contrary, combining synthetic polymers with natural decellularized tissues, provides adequate and tunable mechanical strength and also a native tissue architecture that is highly conducive for cellular honing and proliferation (29, 31, 33–40). Porcine heart valve tissue constructs that were modified with biopolymers improved their *in-vivo* mechanical stability, antithrombogenicity, remodeling, and prevented calcification (31, 40, 41). We previously adapted the hybrid tissue engineering approach to fabricate a planar hybrid biomaterial with multiple applications for cardiovascular reconstruction. We modified BP, which is widely used as a cardiovascular replacement (42, 43) with a biodegradable [polycaprolactone (PCL):chitosan (Ch)] polymer blend to construct a biohybrid material (44). The concept was deduced to a prototype by using non-degradable decellularized BP, with a detergent mixture that removed all the cells, but preserved the extracellular matrix (ECM) architecture. This native ECM core was then overlaid with a matrix of PCL:Ch using an electrospinning technique to deposit nanofibers onto the matrix core in a directionally aligned manner. The hypothesis is that the polymeric mesh restores mechanical function of the pericardia lost due to the decellularization process, and thus is more suitable to evolving cardiovascular mechanical environments. The polymeric mesh will also hypothetically act as a non-thrombogenic, bioactive layer that enables cellular adhesion that precedes cellular infiltration and ultimately, gradual polymer degradation as seen in another scaffold using the same synthetic polymer blend (25). In this study, we report extensive *in-vitro* characterization of this biohybrid material, its mechanical strength using a variety of testing methods, hemocompatibility in blood flow loops with high and low shear stresses and flow disturbances, biocompatibility, and feasibility of this hybrid composite material as a potential cardiovascular replacement material.

## Materials and Methods

### Matrix-Polymer Composite Material (Biohybrid)

Bovine pericardium was sourced from a commercial vendor (Collagen Solutions, Eden Prairie, Minnesota, USA) and the biohybrid material was prepared. The pericardium was decellularized with 2% sodium deoxycholate [D6750; Sigma-Aldrich, St. Louis, Mosby, USA; average molecular weight (MW) 1,200–5,000] for 48 h, followed by 1% sodium deoxycholate for 24 h, and treatment with DNase (D4527; Sigma-Aldrich, St. Louis, Mosby, USA) and RNase (R6513; Sigma-Aldrich, St. Louis, Mosby, USA) for 2 h at 37°C, in a shaker incubator (Model 420; Orbital shaker, Forma Scientific, USA). Acellularity was confirmed by DNA estimation, histology [H&E and 4,6-diamidino-2-phenylindole (DAPI) staining], and scanning electron microscopy (SEM). Twelve percent PCL (Catalog # 440744, Sigma-Aldrich, St. Louis, Mosby, USA, molecular weight 70,000–90,000) and 1% Ch (Catalog # 417963, Sigma-Aldrich, St. Louis, Mosby, USA, molecular weight >1,00,000) blend was prepared in a mixture of 80:20 trifluoroacetic acid (TFA) (L06374, Alfa Aesar, USA) and dichloromethane (DCM) (39116, Alfa Aesar, USA). The polymer solution was then electrospun onto a 10 cm × 12 cm decellularized pericardial core mounted on a rotating mandrel. Polymer fibers (134.68 ± 49.4 nm) were deposited in the circumferential direction on the fibrosa side of the decellularized pericardium, 3 h upto at room temperature, until a thickness of about 70 μm was achieved. The sample was then neutralized in 0.5 M NaOH for 10 min for the free amine of Ch to interact with the decellularized tissue. The sample was then washed in distilled water and preserved in 70% ethanol. Detailed protocols for each method are described in Supplementary Section 1.0.

### Mechanical Integrity of the Decellularized Core and Core-Polymer Interaction

Efficacy of decellularization and its structural integrity were assessed by staining with Masson's trichrome, Verhoeff's Van Gieson, and alcian blue stains. Quantitative estimation of DNA, collagen, elastin, and glycosaminoglycans (GAGs) was performed with DNA Estimation Kit (PureGenome™ Tissue DNA Extraction Kit, Millipore Sigma, USA), Hydroxyproline Assay (MAK008, Sigma-Aldrich, St. Louis, Mosby, USA), Fastin™ Elastin Assay (F2000, Biocolor, UK), and Dimethylmethylene Blue (DMMB) Assay, respectively, using techniques reported earlier (44). Detailed protocols for each step are described in the Supplementary Section 1.1. Polymer-core interface was examined with SEM and molecular interactions were quantified with Fourier transform infrared spectroscopy spectroscopy (FT-IR) and X-ray photoelectron spectroscopy (XPS). FTIR spectra were recorded for PCL, Ch, PCL-Ch blend, decellularized BP, and the biohybrid samples to identify the differences in their functional groups. XPS was used to determine the elemental and chemical composition of each material. Sample preparation techniques and details of each method are provided in Supplementary Section 1.2.

### Core-Polymer Peel and Shear Strength

Strength of the polymer-core interaction was quantified by using two methods—a custom setup to measure the tangential peel force required to delaminate the polymer off the decellularized core and a second experiment in which tubes of the material were prepared and mounted into a flow loop to induce shear stress on the polymer. Details of both the setups are provided in Supplementary Section 1.3. The peel force was plotted against time and the instance of peeling was defined as a point when a sharp change in the force-displacement curve was observed. Peel strength was then calculated as the load imposed tangentially at the time of peel, to the longitudinal cross-sectional area of the material (width × length). In the flow experiments, a shear stress of 15 dynes/sq cm was imposed on the inner walls of the tube made from this material, with a glycerin-water mixture with viscosity equivalent to that of blood. The conduits were exposed to flow for 24 h, after which they were removed and examined with SEM. In another experiment, the conduit was constricted to form a 50% stenosis, to create high velocity flow through the channel and downstream recirculating zones and the experiment was repeated.

### Mechanical Testing

Unconstrained uniaxial testing was performed on untreated BP (*n* = 7), decellularized BP (*n* = 7), and the biohybrid material (*n* = 7). A dog-boned shape die (W: 5 mm × L: 30 mm) was used to cut uniform samples, oriented so the polymer fibers were aligned with the loading direction. Thickness of the samples were measured at multiple regions with a digital caliper and averaged to get an estimated sample thickness. Prior to testing, graphite markers were placed on the sample for optical strain measurements. The sample was mounted in a universal testing machine (Test Resources 100Q, Shakopee, Minnesota, USA) and preconditioned for 10 cycles, with 50% of maximum strain in the elastic region for 10 cycles. The samples were then loaded to failure at a strain rate of 10 mm/min. From the resultant stress-strain data, the uniaxial ultimate tensile strength (UTS), ultimate tensile extensibility (UTE), and the tangential moduli at the upper and lower response regions were calculated and compared between the groups. Constrained uniaxial testing was also performed on a biaxial mechanical testing system (CellScale Biomaterials Testing, Waterloo, Ontario, Canada) with 6 mm × 6 mm samples mounted by using rakes. Sample thicknesses were measured as described above. Four graphite optical markers were placed on the surface of the sample for optical strain measurements, and the samples were immersed in phosphate-buffered saline (PBS) maintained at 37°C throughout testing. All the tissue samples underwent 7 cycles of preconditioning, until hysteresis was absent. The samples were then constrained (fixed) in one direction (axial/circumferential) and then loaded to 10 and 20% strain in the orthogonal direction while recording force and marker positions. The green strains were measured, and the 2^nd^ Piola-Kirchoff stress was calculated. Lastly, samples were loaded equibiaxially by stretching the samples to 10% strain in both the directions uniformly while recording force and optical marker locations to measure green strain and calculate 2^nd^ Piola-Kirchoff stress. Data were fit to a Fung's exponential strain energy function through minimization of an objective function to estimate best fit model parameters. The relationship between the axial and circumferential directions was assessed by using Fung's model coefficients to calculate an anisotropy index (*AI*), where a value of 1 would indicate the tissue was isotropic and values closer to 0 would suggest increasing material anisotropy. Further details can be found in Supplementary Section 1.4.

### Cell Adhesion Study

Porcine mitral valve interstitial cells were isolated and seeded onto the material to assess their attachment to the surface. Though human valve cells would be ideal, we did not have access to these materials. Porcine mitral valves were used to isolate cells to in view of future animal testing of the biohybrid and due to complexity to procure normal human valves. Techniques used to isolate the cells are described in Supplementary Section 1.5. Forty-eight hours after seeding the cells onto the material, the materials were fixed and stained with rhodamine phalloidin and DAPI. Retainment and viability of cells were observed under a microscope (Axioscope A1, Carl Zeiss Microscopy, LLC, USA).

### Hemocompatibility

Hemocompatibility of the bio-hybrid and decellularized BP was assessed by percentage hemolysis assessment assay, clot formation assay, and platelet adhesion assays. Fresh porcine whole blood was collected with ethylenediaminetetraacetic acid (EDTA) (1.6 g/l) and maintained under constant agitation. Porcine blood was used as human blood to the desired volumes was not available. For hemolysis studies, sterile samples were incubated with 5 ml whole blood for 30 min at 37°C. One cc of blood was sampled at baseline and at the end of the experiment and percentage hemolysis was calculated as (free Hb/total Hb) × 100. To assess clot formation, the decellularized BP and the biohybrid samples were incubated in constantly agitated whole blood at 37°C for 30 min. Clot formation was assessed visually. For platelet adhesion assay, platelets were isolated from 30 ml whole blood by centrifugation at 2,000 rpm for 12 min and the supernatant was centrifuged at 5,000 rpm for 15 min. The platelet pellet was resuspended in 2 ml of platelet-poor plasma and 500 μl of platelet suspension was added to the samples. Samples were kept in shaker incubator for 30 min at 37°C at 100 rpm, fixed in formalin for 30 min, and stored in 70% ethanol. SEM was used to image adhered platelets on the surface of these materials.

### Statistical Analysis

Statistical analysis was performed in GraphPad Prism software version 7 (GraphPad Software Incorporation, San Diego, California, USA). All the data were tested for normality by using the Shapiro–Wilk normality test. The untreated BP, decellularized BP, and the biohybrid groups were compared by using the Wilcoxon matched-pairs signed rank test for collagen, GAG, and elastin estimation. For DNA estimation data, a paired *t*-test was used for comparison between the untreated and decellularized groups. For the uniaxial and biaxial mechanical testing, one-way ANOVA was used to test the differences between the three groups tested. All the *p* < 0.05 were considered as statistically significant.

## Results

**Figure 1:**
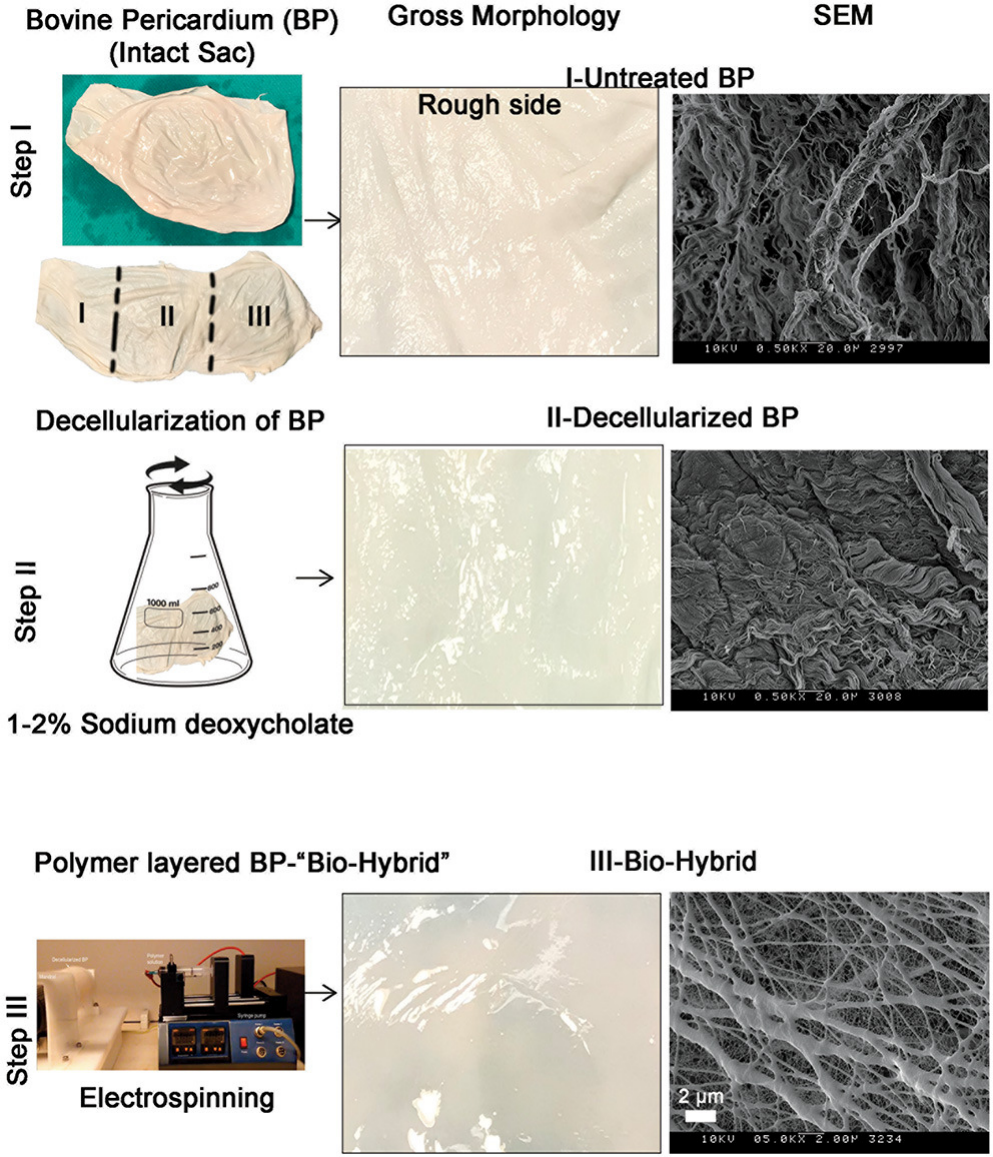
The biohybrid composite fabrication steps by using bovine pericardium (BP). Step I—intact BP is divided into three portions for processing and first portion is left untreated. Right panel shows gross examination and scanning electron microscopy (SEM) image of the untreated BP showing the fibrous side; step II—second portion of the sac is decellularized by using sodium deoxycholate. Right panel shows gross examination of the decellularized BP that shows a blanched appearance due to the detergent and SEM shows the absence of cells on the fibrous side; and step III—coating of polycaprolactone:chitosan polymer layer on the decellularized BP by electrospinning. Right panel shows gross examination of the biohybrid showing a smooth surface after polymer coating and SEM image showing presence of nanofibers on the top surface of BP.

The bio-hybrid scaffold was fabricated by electrospinning PCL-Ch nanofibers onto the rough side of decellularized BP. The three groups of materials used for *in-vitro* experiments (untreated BP, decellularized BP, and the biohybrid) were characterized by gross morphology and surface characteristics, as shown in Figure 1. The untreated BP had a fibrous appearance both in the gross observation and electron microscopy, which also showed the presence of cells (Figure 1—Step I). The decellularized BP had fibrous structure on electron microscopy and in gross morphology and appeared blanched due to the treatment with detergent. Gross observation of the biohybrid showed a smooth glistening surface and the presence of polymer nanofibers in SEM (Figure 1—Step III). The detailed characterization of the polymer nanofibers has been reported previously (44).

### Decellularization of BP and ECM Characterization

**Figure 2:**
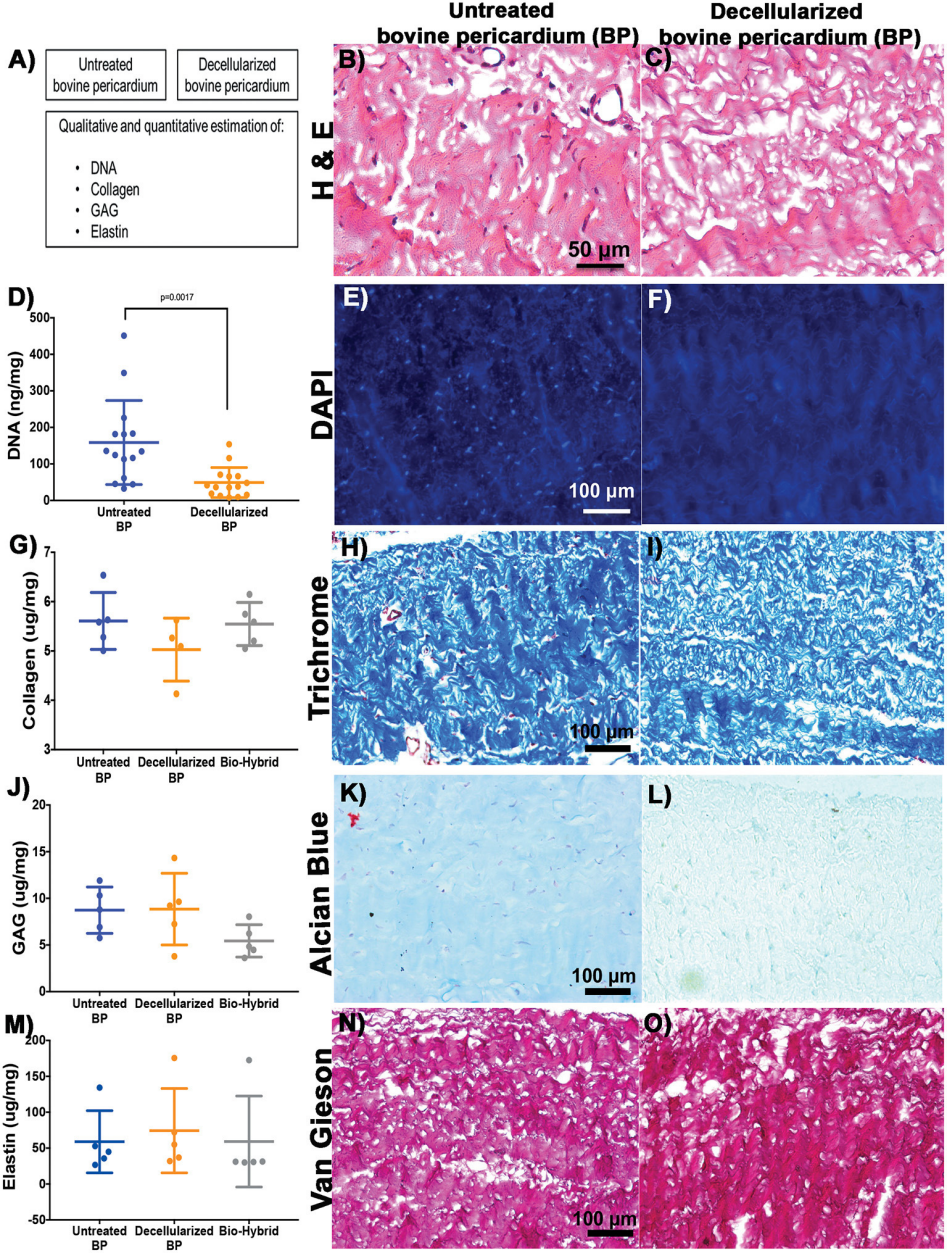
Characterization of the untreated BP and decellularized BP by using histopathology and biological estimation of extracellular matrix proteins. **(A–C)** Schematic representation of the characterization and H&E staining showing absence of cells in the decellularized BP. **(D–F)** Quantification of total DNA showing significant reduction in the decellularized BP and 4,6-diamidino-2-phenylindole (DAPI) staining showing absence of DNA in the decellularized BP. **(G–I)** Quantification of collagen showing no reduction of collagen in the decellularized BP and the biohybrid and trichrome staining showing presence of collagen represented by blue color in the decellularized BP. **(J–L)** Quantification of glycosaminoglycans (GAGs) showing no significant reduction of GAGs in the decellularized BP and the biohybrid and Alcian blue staining showing presence of GAG represented by cyan color in the decellularized BP. **(M–O)** Quantification of elastin showing no significant reduction of GAGs in the decellularized BP and the biohybrid and Verhoeff's Van Gieson staining showing presence of elastin represented by black fibers in the decellularized BP.

Decellularization was a prerequisite for the fabrication of the bio-hybrid scaffold and decellularized BP was analyzed for acellularity and ECM integrity as shown in schematic Figure 2A. H&E and DAPI staining showed the absence of nuclei in the decellularized BP compared to the untreated BP (Figures 2A–F). DNA content significantly decreased after decellularization from 158.6 ± 115.1 to 49.06 ± 41.1 ng/mg (*p* < 0.05) in the untreated BP and decellularized BP, respectively (Figure 2D). Major ECM components were preserved after decellularization and after biohybrid fabrication as shown by histology and ECM assays (Figures 2G–K). Collagen did not significantly decrease after decellularization and the hybrid tissue fabrication (Figure 2G). Collagen was quantified at 5.6 ± 0.58, 5.0 ± 0.64, and 5.5 ± 0.44 μg/mg in the untreated BP, decellularized BP, and the bio-hybrid, respectively. Collagen retention was also seen in trichrome staining indicated by blue fibrils, as shown in Figures 2G–I. Similarly, elastin and GAG did not significantly decrease (Figures 2J–O) after fabrication of the bio-hybrid. GAG and elastin concentration were 58.9 ± 43.31, 74.29 ± 58.79, and 59.14 ± 63.39 μg/mg and quantified as 8.73 ± 2.48, 8.84 ± 3.83, and 5.43 ± 1.7 μg/mg in the untreated BP, decellularized BP, and the bio-hybrid, respectively. Figures 2B–F show the reduction of nuclei in the decellularized BP in comparison to the untreated BP, both quantitatively and qualitatively. Large variability was observed in the ECM protein components due to the heterogenicity in the pericardia between animals, and the relatively smaller sample size. Such heterogeneity was reported by other studies as well (43, 45).

### Polymer-Tissue Interface Characterization

**Figure 3:**
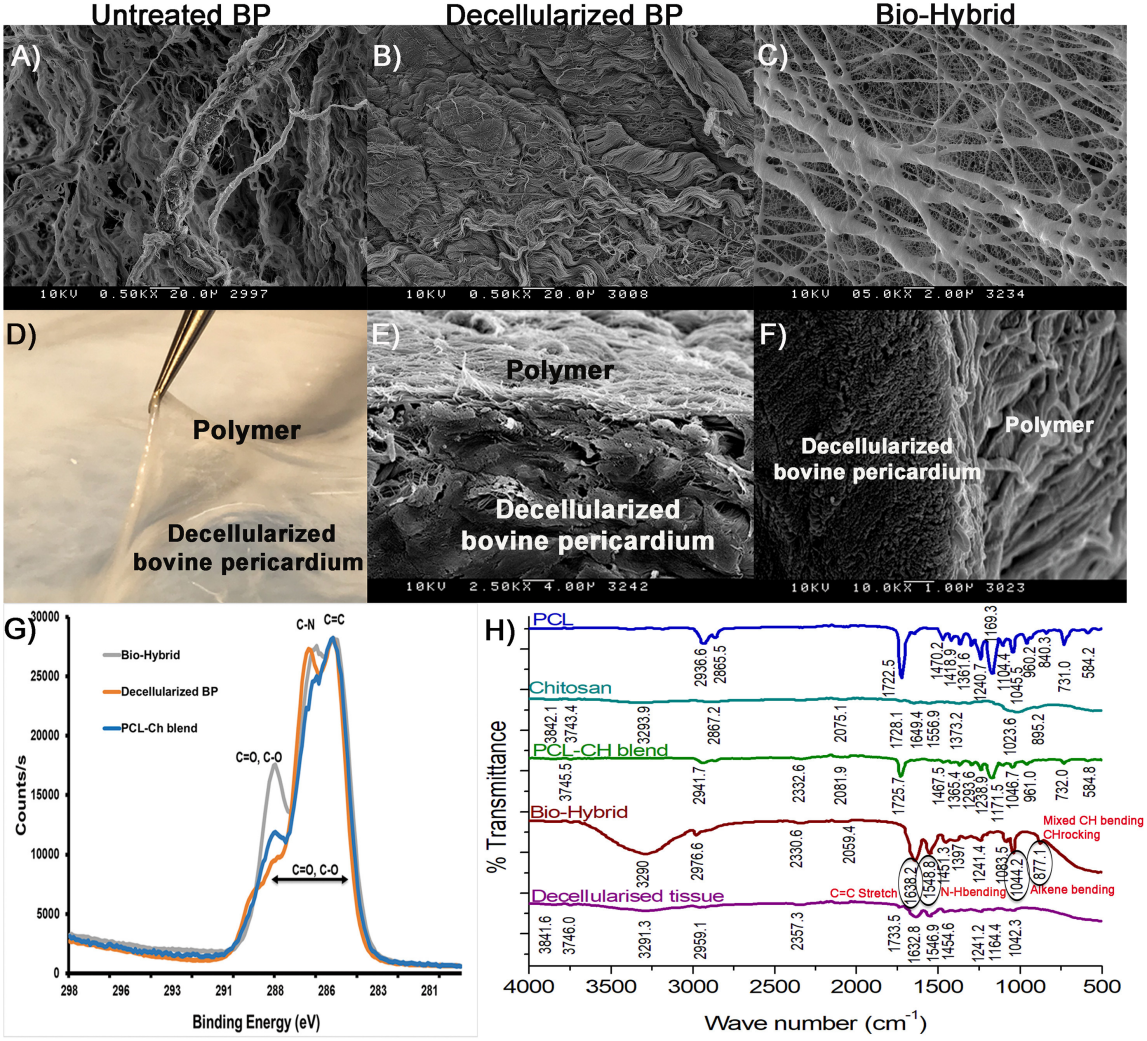
Polymer-tissue interface characterization in the biohybrid composite. **(A–C)** SEM images of the untreated BP, decellularized BP, and the biohybrid showing the presence of cells in the untreated BP, absence of cells in decellularized BP, and coating of electrospun polymer nanofibers on the decellularized BP in the biohybrid composite, **(D–F)** photograph of the biohybrid composite showing lifting of polymer from the decellularized BP followed by SEM images of the cross-sectional views of the biohybrid showing presence of polymer fibers at the interface without separation at the interface, **(G)** carbon scanning of the biohybrid, decellularized BP, and polycaprolactone:chitosan blend by X-ray photoelectron spectroscopy showing difference in binding energy corresponding to peaks C=C, C=O, C-O, and C-N in the biohybrid, and **(H)** Fourier transform IR spectroscopy of polycaprolactone, chitosan, blend, the biohybrid, and decellularized BP showing unique peaks in the biohybrid corresponding to changes in the C=C, C=O, N-H, and C-H groups.

Scanning electron microscopy images of the untreated BP, decellularized BP, and the biohybrid are shown in Figures 3A–C. Bovine pericardial surface depicts cells integrated with the fibers (Figure 3A), whereas decellularization removed the cells while preserving the matrix architecture (Figure 3B). The biohybrid surface depicts nanofibers overlaid on the core and covering it (Figure 3C). The individual polymer blend nanofibers electrospun on aluminum foil are approximately 134.68 ± 49.4 nm (data not shown here) that fuse during the hybrid tissue preparation to form thicker fibers. Figures 3D–F depict the interface between the polymer and the matrix core and a SEM image of the cross-section depicting adherence of the polymer to the underlying matrix core. XPS results of each of the materials are shown in Figure 3G, which provide elemental analysis in a quantitative manner. In the bio-hybrid material, new peaks corresponding to C=O, C-O, and C-N were observed, which were not seen in the decellularized BP alone or the polymer blend alone. FTIR data in Figure 3H depict the spectra of the newly formed chemical bonds between the polymer and the underlying ECM. Two new peaks at 1548.8 and 1638.2 cm^−1^ corresponding to the amide groups and two new peaks at 877.1 and 1044.2 cm^−1^ corresponding to C–O–C stretch were measured.

### Peel Strength of the Bio-Hybrid

**Figure 4:**
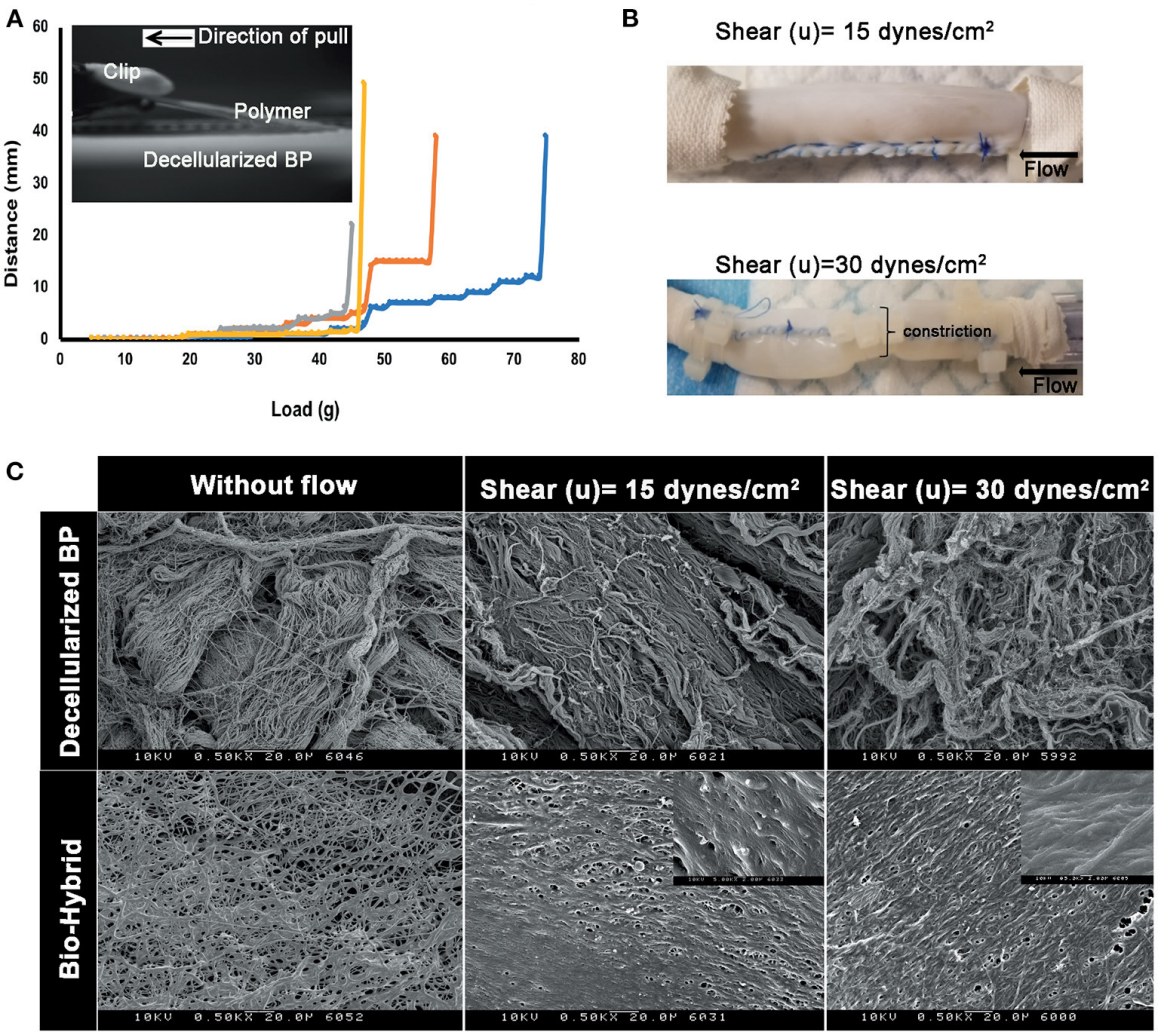
Peel strength of the polymer in the biohybrid. **(A)** Peel profile of the biohybrid composite showing the load measured to peel the polymer in the biohybrid from the decellularized BP by using a customized setup where the polymer was peeled by using a clip attached to a weight hanger and a pulley, **(B)** image of the biohybrid composite conduit subjected to low shear and high shear (a portion of sample constricted to about 50% of original diameter) experienced by a normal artery (15 dynes/cm^2^) in a continuous flow loop, and **(C)** SEM images of the decellularized BP samples subjected without any shear (without flow), low (15 dynes/cm^2^) and high shears (30 dynes/cm^2^) showing extent of damage of the extracellular matrix fibers due to the shear. SEM images of the biohybrid samples subjected without any shear (without flow), low and high shears showing non-delamination and smoothening of the polymer surface when subjected to shear.

Results of the peel strength and shear-induced delamination experiments are shown in Figure 4. The load required to peel the polymer from the surface of the matrix core is given in Figure 4A from the four distinct bio-hybrid samples ranging from 40 to 75 g, with an average force of 56.13 ± 11.87 mN/mm^2^ g required to delaminate the polymer. The samples induced with two different shear stress conditions are shown in Figure 4B, depicting the cylindrical sample with and without a constriction. SEM images of the luminal surfaces of the decellularized BP and the biohybrid, with and without flow, are shown in Figure 4C and Supplementary Figure 1. At 15 and 30 dynes/cm^2^ of shear stress, disarray of the decellularized fibers was observed, with higher damage associated with higher shear rates. In the bio-hybrid, the polymer nanofibers did not peel or disrupt, but formed a more uniform layer on the tissue, aligned along the flow direction. In either case, the polymer was not delaminated from the underlying pericardium.

### Mechanical Testing of the Biohybrid Composite Material

**Figure 5:**
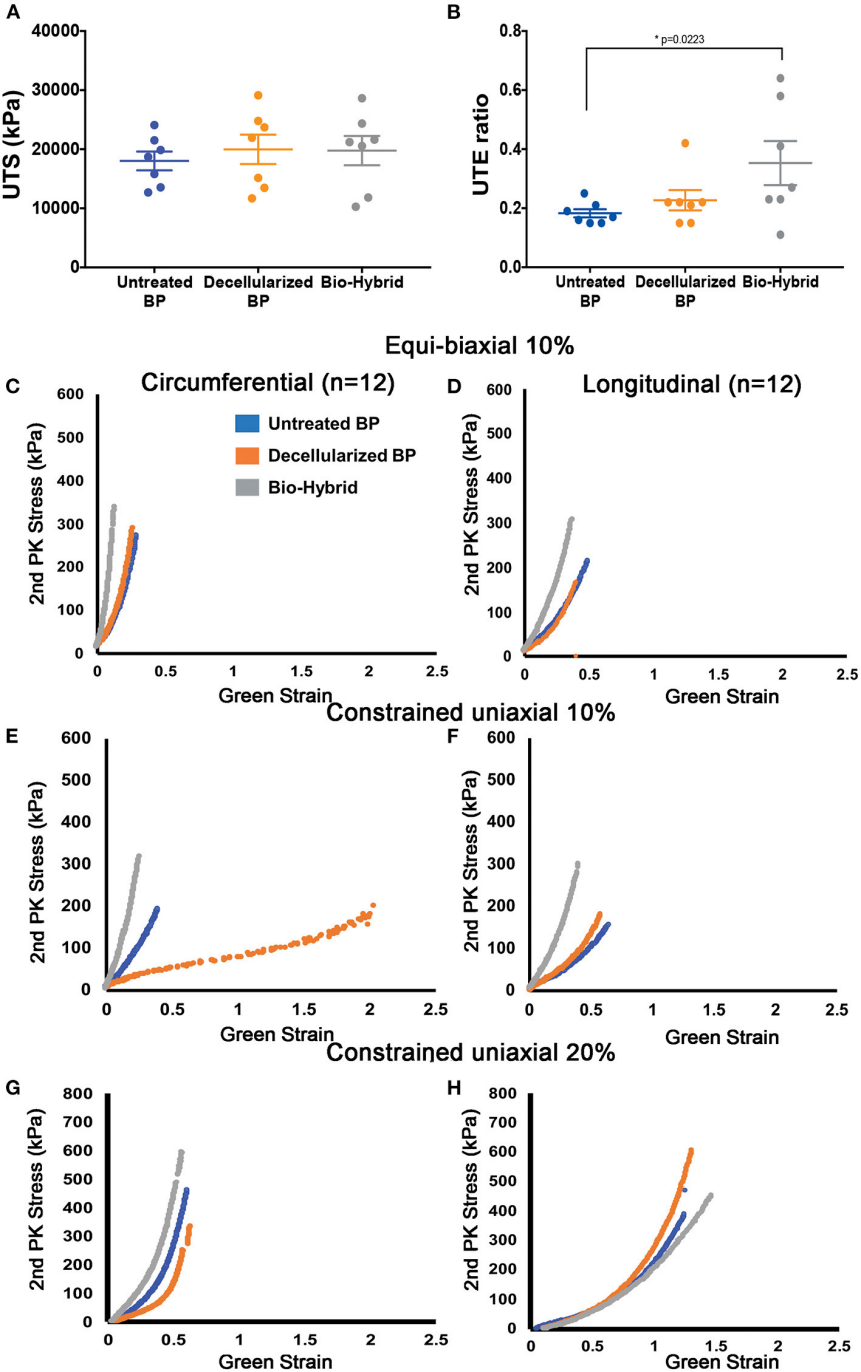
*In-vitro* mechanical properties of the untreated BP, decellularized BP, and the biohybrid composite. **(A,B)** Uniaxial tensile strength (kPa) and uniaxial tensile extensibility ratio of the materials showing no difference in the tensile strength and a significant increase in the extensibility in the biohybrid, **(C,D)** equibiaxial (10%) testing of the three groups showing a stiffer response of the biohybrid in both the circumferential and longitudinal directions and similar response of the fresh BP and decellularized BP, **(E,F)** step biaxial testing with 10% strain in the direction of testing showing stiffer response of the biohybrid than the other two groups in both the directions whereas the decellularized BP is more compliant in the circumferential direction, and **(G,H)** step biaxial testing with 20% strain in the direction of testing showing stiffer response of the biohybrid in the circumferential direction followed by the untreated BP and decellularized BP and longitudinal direction shows stiffer response of the decellularized BP with absence of aligned polymer nanofibers in the biohybrid.

**Figure 6:**
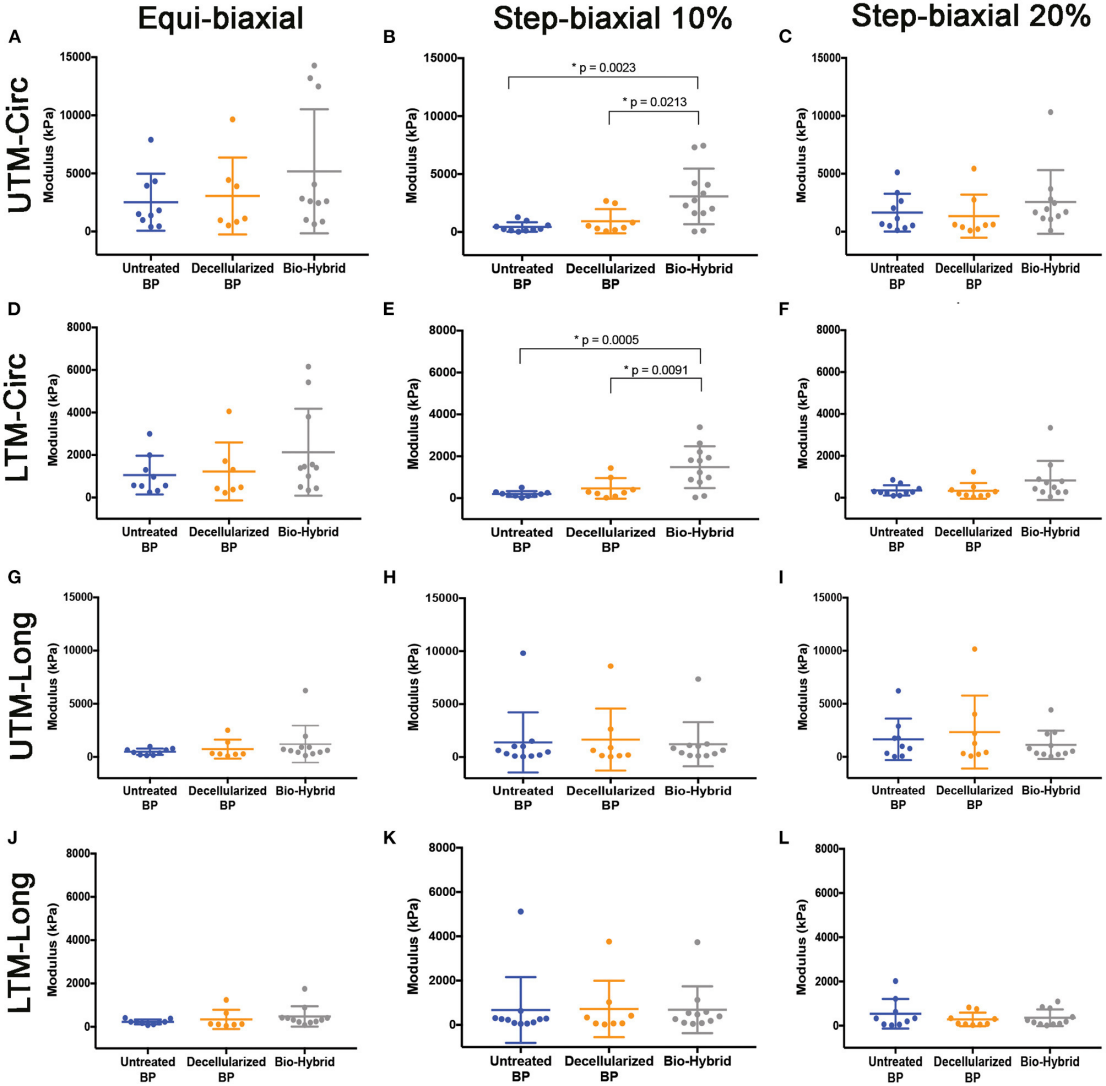
Biaxial upper tangent modulus (UTM) and lower tangent modulus (LTM) of the untreated BP, decellularized BP, and the biohybrid samples from equibiaxial and step biaxial tests. **(A–C)** UTM in the circumferential direction; **(D–F)** LTM in the circumferential direction; **(G–I)** UTM in the longitudinal direction; and **(J–L)** LTM in the longitudinal direction. Data are represented as mean ± SD. *p* < 0.05 was considered to be statistically significant.

Unconstrained uniaxial mechanical testing of the untreated BP, decellularized BP, and the bio-hybrid samples did not show any difference in the ultimate tensile strength (untreated BP: 18,000 ± 4,200 kPa, decellularized BP: 20,000 ± 6,600 kPa, and the bio-hybrid: 20,000 ± 6,600 kPa), as shown in Figure 5A. However, there was a significant increase in the tensile extensibility (Figure 5B) of the biohybrid compared to the untreated BP (untreated BP: 18 ± 3.7%, decellularized BP: 23 ± 9%, and the bio-hybrid: 35 ± 2%). While constraining the samples by 10% in the axial direction and stretching in the circumferential direction to 10%, the bio-hybrid material had an increased upper and lower tangential moduli compared to BP and decellularized BP [bio-hybrid upper tangent modulus (UTM) and lower tangent modulus (LTM) = 3,071 ± 693 and 1,481 ± 289 KPa; decellularized BP UTM and LTM = 930 ± 370 and 467 ± 174 KPa; and untreated BP UTM and LTM = 435 ± 129 and 200 ± 42 KPa], but was not evident when constraining the sample by 20% or when constrained in the circumferential direction and loaded in the axial direction for either condition (Figure 6). Results from equibiaxial testing are shown in Figures 5C,D, which highlight the native intraspecimen variability in mechanical response of BP that leads to samples with large variations in mechanical properties, which limited statistical findings. Supplementary Figure 3 depicts the biaxial stress strain curves for all the samples tested. The Fung model was appropriately fitted to each individual data sets with an average root mean square error (RMSE) of 9.49 ± 2.53 kPa, 11.11 ± 9.97 kPa, and 13.74 ± 15.22 kPa for the untreated BP, decellularized BP, and the bio-hybrid materials, respectively. Decellularization of BP trended toward an increase in material anisotropy (AI: 0.32 ± 0.15) compared to untreated BP (AI: 0.55 ± 0.21), although not statistically significant. Deposition of the PCL/Ch nanofibers on the biohybrid appeared to partially restore the AI of the material (AI: 0.45 ± 0.25) to that of BP prior to decellularization, although not statistically significant (Table 1).

**Table 1:** Fung model parameters and anisotropy metric.

	***c***	***a_**1**_***	***a_**2**_***	***a_**3**_***	***RMSE (kPa)***	***AI***
Fresh BP	528.9 ± 650.22	11.56 ± 24.19	7.06 ± 15.89	1.29 ± 2.95	9.49 ± 2.53	0.55 ± 0.21
Decellularized BP	19058.2 ± 46137.75	4.12 ± 4.15	1.17 ± 1.03	0.26 ± 0.22	11.11 ± 9.79	0.32 ± 0.15
Biohybrid	278.42 ± 302.94	25.33 ± 48.76	5.78 ± 7.83	1.92 ± 3.31	13.74 ± 15.22	0.45 ± 0.25

*Fung exponential model parameters and anisotropy index (AI) for the each experimental group. RMSE is the root mean square error. Data are presented as mean ± SD*.

### Biocompatibility and Hemocompatibility of the Biohybrid

**Figure 7:**
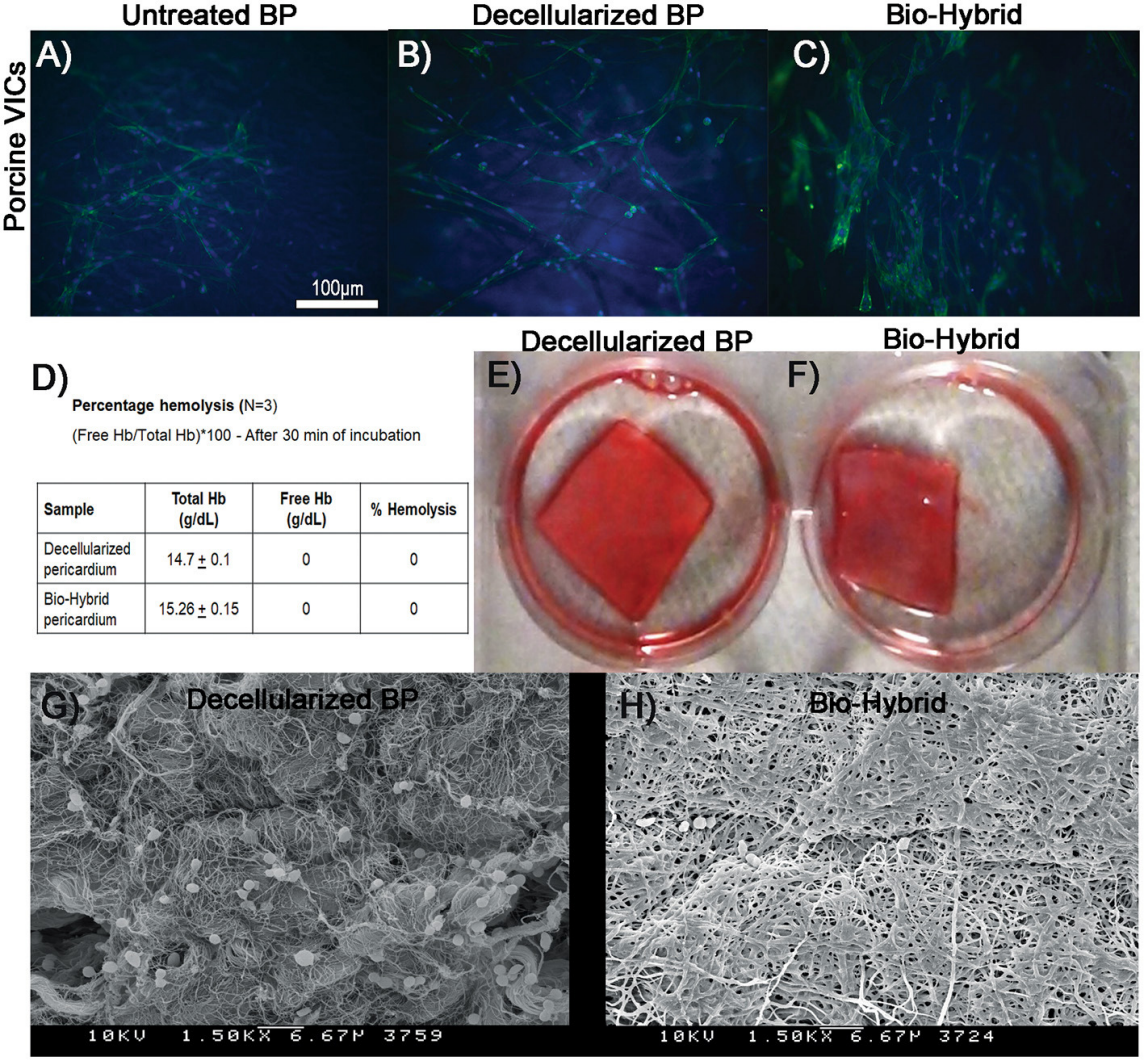
*In-vitro* biocompatibility and hemocompatibility studies of the decellularized BP and the biohybrid composite. **(A–C)** *In-vitro* biocompatibility of the untreated glutaraldehyde-fixed BP, decellularized BP, and the biohybrid by cell adhesion assay. Cell adhesion of porcine valve interstitial cells showing good attachment on the decellularized BP and the biohybrid samples compared to untreated glutaraldehyde-fixed BP, **(D)** percentage hemolysis of cells on the decellularized BP and the biohybrid showing no hemolysis on both the samples when incubated with fresh porcine blood for 30 min, **(E,F)** clot formation assay showing no clots on both the decellularized BP and the biohybrid samples after 30 min of incubation with blood, and **(G,H)** SEM images of the decellularized BP and the biohybrid incubated with platelets showing very minimal platelet adhesion on the biohybrid compared to the decellularized BP.

The *in-vitro* biocompatibility of the bio-hybrid composite is shown in Figures 7B,C where the bio-hybrid and decellularized BP showed similar and better attachment of porcine valve interstitial cells whereas the untreated BP (Figure 7A) showed less attachment of cells seen visually on these samples. Figures 7D–H show *in-vitro* hemocompatibility of the bio-hybrid by using three different tests. The bio-hybrid and the decellularized BP samples did not show any hemolysis (0 g/dl) of cells upon agitating with fresh blood as shown in Figure 7D. Clots did not form on the decellularized BP and the bio-hybrid samples demonstrating unchanged hemocompatibility of the bio-hybrid with the addition of polymer to the decellularized core (Figures 7E,F). Additionally, there was minimal platelet adhesion on the bio-hybrid in comparison to the decellularized BP core as shown in the SEM images (Figures 7G,H).

## Discussion

Data from this study demonstrate the feasibility of the bio-hybrid composite for use as a cardiovascular tissue substitute. Combining a non-reactive, base material such as decellularized ECM with native three-dimensional structure, with a reactive and slowly biodegrading polymeric covering, it provides a new approach for potential *in-situ* cardiovascular tissue engineering. Though this study does not demonstrate *in-vivo* results in support of this claim, the *ex-vivo* measurements demonstrate good mechanical strength of the layered scaffold, biocompatibility that is evident from cellular adhesion and viability, and hemocompatibility from minimal platelet adhesion in blood flow loops. Altogether, this *ex-vivo* data demonstrate the potential feasibility of this new approach.

The choice of the two materials used in this study builds upon current clinical knowledge supporting the use of these materials independently in the cardiovascular system. Glutaraldehyde-treated BP is currently the gold standard in cardiac surgery, as it has good mechanical strength and stability, adequate shelf-life, and non-thrombogenicity (42, 46–48). Despite these favorable characteristics, structural deterioration and calcification are observed, primarily due to the host immune response to the glutaraldehyde fixative and the cells within the tissue (6, 48, 49). This issue was addressed previously, by decellularization of the BP, reducing the total DNA content, and maximizing the native ECM protein content such as collagen, elastin, and GAGs (50–52). Despite these measures, the mechanical strength upon decellularization is lessened, with uncontrolled material anisotropy from changes in the fiber architecture and alignment (53). Structural degradation of the decellularized material is a risk, decreasing its use in the cardiovascular system. In the bio-hybrid material, under uniaxial loading, increased material extensibility along the fiber direction was observed, without significant bulk stiffening. When constrained in the axial direction with a 10% stretch and loading in the circumferential direction (fiber direction), the material appeared to stiffen. When the axial stretch was increased to 20%, this stiffening was not observed, suggesting that the polymer fibers were either reoriented to bear load or damaged at these loads. It is likely that reorientation had occurred at higher loads, as we did not observe any damage to the polymeric layer. The *in-vitro* scaffold stability and degradation of the decellularized BP and the bio-hybrid were previously studied by us and demonstrated that both the materials did not degrade significantly in stimulated physiological conditions (37°C, pH 7.0) for up to 30 days (44, 54). Also, the ultimate tensile strength of human cardiovascular tissue in uniaxial tension ranges from 1 to 3 MPa and the bio-hybrid scaffolds with the 70 μm thick polymer layer is significantly stronger as seen in Figure 5 (55, 56). With a degradation of about 7% in 30 days [from our previous study (54)], we assume that the biohybrid will most likely remain stable and allow ECM remodeling when implanted *in vivo*, similar to other cardiovascular tissue replacement biomaterials (21, 57, 58). The *in-vivo* degradation is likely to differ from *in-vitro* conditions due to the complex interplay of immune response, expression of matrix degradation enzymes, and macrophage expression that is absent in *in-vitro* experiments (58). It is known that synthetic biomaterials are known to experience chronic inflammatory response and the decellularized tissues are less immunogenic, but are weaker and more susceptible to structural degradation. Bio-hybrid scaffold material that has a non-degradable bovine pericardial core (degradation time about 10 years) with a biodegradable polymer coating (PCL degradation time is about 2 years) would possibly experience intermediary inflammatory response and provide enough duration for matrix remodeling as cardiovascular substitute material similar to the other hybrid scaffolds (23, 25, 58–61). Previous work has shown that PCL:Ch vascular grafts, by using similar concentrations of the polymers, showed good vascular remodeling (25); however, this has not been studied as a cardiovascular replacement tissue. FTIR and XPS data in this study demonstrate that the polymeric layer also formed bonds with the underlying collagen proteins in the decellularized matrix, which may promote anchoring of both the layers and reorientation under loading. Hydrogen bond formation between the hydroxyl group of Ch and the ester group of PCL were observed, which, in turn formed amide linkage between the PCL:Ch and decellularized core as shown in XPS and FTIR (Figure 3).

Polycaprolactone was chosen, as it is Food and Drug Administration (FDA)-approved synthetic polymer with tunable mechanical properties, hydrophobicity that inhibits platelet attachment and slow degradation making it suitable for use in the cardiovascular system. However, the inherent hydrophobicity does not enable cellular attachment and, thus, promote *in-situ* host tissue engineering of the scaffold (59). Ninety percent deacetylated Ch at a very low concentration (1%) was added to PCL, increasing its hydrophilicity to an extent that cellular infiltration and survival may be possible. Ch has a structure similar to native GAGs, which may provide the moieties required for cellular adhesion and further infiltration. Recently, PCL-Ch small-diameter vascular grafts, with high concentrations of Ch, were used successfully in sheep up to 6 months (25).

The polymer could be overlaid on the matrix in several ways, but electrospinning provided an approach that can create a 3-dimensional surface topography that would enable cellular attachment (44, 54). Electrospinning was preferred than commonly used dip coating for polymer-tissue combination due to the potential damage from organic solvents (41, 55). Additionally, the polymer deposition on the BP in this study in its native 3D form negates the risk of degeneration and calcification that has been otherwise seen previously in cryopreserved tissues (56, 62). The coating of a biocompatible polymer on decellularized tissue would act as an immune barrier to antigenic proteins present after decellularization that has shown to be beneficial to improve cellular adhesion and mechanotransduction that, in turn, improves *in-vivo* remodeling (31, 40, 41). Nanofiber-microdimension architecture has been shown by others to improve cellular adhesion with stronger attachment in comparison to the smoother biomaterial surfaces due to higher surface to volume ratio and similar 3D topography of natural tissues leading to enhanced deposition of ECM proteins (63, 64). We, thus, chose to electrospun PCL:Ch nanofibers (134.68 ± 49.4 nm) to mimic the surface similar to natural ECM fibers that fuse to form around 300 to 500 μm fibers during the bio-hybrid processing that allowed excellent cellular attachment and alignment on the bio-hybrid compared to the decellularized BP and untreated BP (Figure 7). While this investigation was limited to this specific polymeric blend, other polymers that are combined with small molecules can be used in the future for specific targeted outcomes.

With the specified modifications to the pericardial preparation, *in-vitro* biocompatibility studies showed better cellular attachment onto the bio-hybrid (Figure 7). The cellular attachment is likely from the hydrophilicity that the PCL:Ch blend imparts, which is not seen in the PCL-based biomaterials (59). The biohybrid material also exhibited adequate *in-vitro* hemocompatibility with no hemolysis or clot formation, and minimal platelet attachment when the material was agitated in blood (Figure 7). The hemocompatibility of the polymeric blend can be attributed to the hydrophobicity of the PCL and smooth surface of the polymer that does not allow the platelets to adhere. Such behavior was shown by others using this PCL:Ch combination for vascular tissue engineering (25, 65). In a dynamic *in-vitro* setup, the polymer layer did not delaminate when subjected to shear equivalent to that of a normal artery in a closed flow loop setup (Figure 4), suggesting its use as a cardiovascular replacement material. The lack of polymer delamination in the flow loop setup correlated with data from the peel strength experiments, which confirmed that adhered polymer layer in the bio-hybrid could withstand physiological flow (Figure 4). The biocompatible and hemocompatible bio-hybrid material with a time-bound degradable polymer layer (since PCL has a degradability of around 2 years *in vivo*) is hypothesized to provide a favorable interface to attract cells and at the same time provides matrix stability over the first few weeks to months after implantation that may redirect it toward remodeling and not fibrosis.

From a translational perspective, the proposed bio-hybrid composite material has several benefits, with a strong decellularized core that provides the mechanical strength for the tissues, while the degradable polymeric sacrificial layer that can enable anisotropy, acute and short chronic immune response until degradation, and cellular honing is achieved. This ensures that as the scaffold remodels, adequate mechanical strength to sustain the hemodynamic forces is available. Thus, the proposed material could be used in high pressure environments as well, such as for patching the carotid artery after endarterectomy, as arterial grafts in children, and potentially as valve leaflets as well. The clinical relevance of this material as a patch or shaped into different implants and their functional efficacy requires long-term studies in animals, which is our next step.

## Study Limitations

As with any experimental study, some limitations should be considered. The materials and methods used in this study are off-the-shelf materials and custom build devices and, thus, are not built per good manufacturing practices (GMPs) standards. Thus, a higher degree of variability between samples is observed. Moreover, there is inter- and intraspecimen variability in native BP, which makes batch processing inconsistent and limits statistical findings of material responses. In future, the most homogenous region of BP will be considered for making the bio-hybrid. Secondly, the *in-vivo* large animal studies are needed to study the immune response along with long-term efficacy and remodeling. Lastly, the efficacy of the bio-hybrid can be better explained when compared to commercially available glutaraldehyde fixed and decellularized tissues that are used clinically.

## Conclusion

The proposed bio-hybrid approach to combine a natural decellularized pericardium, with polymeric nanofibers, has adequate mechanical strength, biocompatibility and hemocompatibility, making it a potentially translatable cardiovascular tissue substitute.

## Data Availability Statement

The original contributions presented in the study are included in the article/Supplementary Material, further inquiries can be directed to the corresponding author.

## Author Contributions

JM worked on the idea generation, experimental design, execution of experiments, data analysis, and manuscript preparation. DX, VW, AA, BL, and DC contributed to the experimental work and data analysis of this study. MP contributed to the experimental design, data validation, and editing of the manuscript. All authors edited and approved the final version of the manuscript.

## Funding

This study was supported by grants from the American Heart Association (19POST34380522) and an infrastructure support from the Carlyle Fraser Heart Center at Emory University Hospital Midtown.

## Conflict of Interest

JM and MP are co-inventors of a patent application relevant to this technology, whose rights are assigned to Emory University. MP owns stock in Nyra Medical, and is an officer of this company. This entity did not have any role in this study, nor did it sponsor or review it. MP has received personal consulting fees from Heart Repair Technologies, which again did not have any role in this work. The remaining authors declare that the research was conducted in the absence of any commercial or financial relationships that could be construed as a potential conflict of interest.

## Publisher's Note

All claims expressed in this article are solely those of the authors and do not necessarily represent those of their affiliated organizations, or those of the publisher, the editors and the reviewers. Any product that may be evaluated in this article, or claim that may be made by its manufacturer, is not guaranteed or endorsed by the publisher.

## Abbreviations

SEM: scanning electron microscopy
FT-IR: Fourier-transform infrared spectroscopy
XPS: X-ray photon spectroscopy
GAG: glycosaminoglycans
DAPI, 4: 6-diamidino-2-phenylindole
PCL: polycaprolactone
Ch: chitosan
H&E: hematoxylin and eosin
Glut: glutaraldehyde
BP: bovine pericardium
ECM: extracellular matrix
Glut-BP: glut fixed untreated BP.
