# Supplementary material for: A Biohybrid Material With Extracellular Matrix Core and Polymeric Coating as a Cell Honing Cardiovascular Tissue Substitute
Source: Front Cardiovasc Med. 2022 Mar 24;9:807255. doi: 10.3389/fcvm.2022.807255 (PMC8987446; doi:10.3389/fcvm.2022.807255)
Supplement: Supplementary file 1 [file Data_Sheet_1.docx]

Supplement material

**Section 1.0 Preparation of matrix-polymer hybrid composite material:**

**Bovine pericardial (BP) sample preparation and decellularization:** Intact bovine pericardial sacs were purchased from Collagen Solutions in ice cold buffer solution, pH 7.4 without Ca^2+^ and Mg^2+^ and transferred to 70% ethanol. The sac was laid down as a flat sheet by cutting in between the pericardial sternal ligaments and then divided into three experimental groups: a) untreated BP- no processing, b) decellularized BP- decellularization with detergent and enzymes and c) Bio-Hybrid -polymer layered decellularized BP. The untreated BP was fixed with 0.625 % buffered glutaraldehyde solution for implantation studies and *in-vitro* biological studies. The groups (b) and (c) were decellularized using 2% sodium deoxycholate (D6750, Sigma-Aldrich, USA, average MW 1200-5000) for 48 hrs followed by 1% sodium deoxycholate for another 24 hrs and treatment with DNase (D4527; Sigma-Aldrich, MO, USA) and RNase (R6513; Sigma-Aldrich, MO, USA) for 2 hrs at 37ºC with continuous stirring in a shaker incubator (Model 420; Orbital shaker, Forma Scientific). The untreated BP and the decellularized BP were stored in 70% ethanol until further use.

**Confirmation of decellularization of BP:** Acellularity of the decellularized BP in comparison to the untreated BP was confirmed by DNA estimation, histology (hematoxylin and eosin (H&E) and 4,6-diamidino-2-phenylindole (DAPI) staining), and Scanning Electron Microscopy (SEM). DNA was extracted using PureGenome™ Tissue DNA Extraction Kit, Millipore Sigma, USA. Extraction was followed according to manufacturer and DNA was estimated spectrophotometrically at 260 and 280 nm using Nanodrop (Thermo Fisher Scientific, USA). For histopathology, 5 μm paraffin embedded sections were stained and imaged using a fluorescent microscope (Axioscope A1, Carl Zeiss Microscopy, LLC) to confirm decellularization. SEM samples were fixed in 2.5 % glutaraldehyde, serially dehydrated up to 100% ethanol and sputter-coated with Gold/Palladium alloy. High resolution SEM (Topcon DS 130F) images were taken to confirm acellularity and extracellular matrix integrity in the decellularized samples.

**Extracellular matrix integrity of decellularized core:** Preservation of extracellular matrix after decellularization was assessed by histology to confirm collagen, elastin and GAGs by trichrome, verhoeff’s van gieson and alcian blue staining respectively and estimation of collagen, elastin and GAGs using hydroxyproline assay kit (MAK008, Sigma-Aldrich, USA), Fastin™ Elastin Assay kit (F2000, Biocolor, UK) and DMMB assay respectively. The estimation of collagen and elastin were followed according to the manufacturer’s instructions and the protocol for GAGs was as described previously(1). Hydroxyproline, α-elastin and GAGs were estimated at 560, 513 and 540 nm.

**Preparation of matrix-polymer hybrid composite material (Bio-Hybrid):** Bio-Hybrid scaffold was fabricated as previously described(1). 12% polycaprolactone-PCL (440744, Sigma-Aldrich, USA, MW 70,000-90,000) was blended with 1% chitosan-Ch (417963, Sigma-Aldrich, USA, MW > 1,00,000) in a mixture of trifluoracetic acid (TFA; L06374, Alfa Aesar, USA) and dichloromethane (DCM; 39116, Alfa Aesar, USA) in the ratio of 80:20. The polymer blend was then electrospun onto the decellularized BP along the circumferential direction of decellularized BP for three hours using a customized electrospinning device (Collector MSK-ESDC-80-450; High voltage supply MSK-ESPS-TE4020; Syringe pump EQ-500SP purchased from MTI Corporation, USA). Electrospinning was performed at room temperature using a 22G blunt stainless-steel needle, voltage of 15 kV, 0.5 mL/h flow rate, and 15 cm between the needle-tip and collector. The polymer was deposited on the decellularized BP by securing it onto the mandrel in a hydrated form on a wet absorbent pad. The samples were neutralized in 0.5M NaOH for 10 min to fully regenerate the free amine form of chitosan so that it can interact with the tissue. The polymer layered decellularized BP (Bio-Hybrid) was washed in distilled water and then preserved in 70% ethanol for further use.

**Section 1.1 Estimation of extracellular matrix proteins in the decellularized and composite materials:**

In addition to the acellularity, the preservation of the extracellular matrix after decellularization was assessed by a) histology to confirm presence of collagen, elastin and glycosaminoglycans (GAGs) by trichrome, verhoeff’s van gieson and alcian blue staining, respectively and b) estimation of collagen, elastin and GAGs using hydroxyproline assay kit (MAK008, Sigma-Aldrich, USA), Fastin™ Elastin Assay kit (F2000, Biocolor, UK) and DMMB assay, respectively. The protocols for collagen and elastin were followed according to the manufacturer’s instructions and the protocol for GAGs was as described previously(1). For collagen estimation, 10 mg of sample was digested in 12 M concentrated hydrochloric acid (HCl) to hydrolyze at 120 °C for 3 hrs. To the hydrolyzed product, chloramine oxidation buffer and (Dimethylamino)benzaldehyde (DMAB) were added. Finally, the hydroxyproline concentration was determined by the reaction of oxidized hydroxyproline with DMAB that resulted in a colorimetric product, proportional to the hydroxyproline content that was measured spectrophotometrically at 560 nm. The amount of collagen in the samples was determined from the standard curve of collagen. For elastin estimation, 40 mg of sample was heated at 100 °C for one hour with 0.25M oxalic acid to convert the native hydrophobic elastin into a water-soluble derivative (α-elastin). The α-elastin was then precipitated using precipitating reagent and quantitatively measured by amount bound to the dye TPPS (5,10,15,20-tetraphenyl-21H,23H-porphine tetra-sulfonate) at 513 nm. The amount of elastin was then determined from the standard curve of elastin. For GAG estimation using the DMMB assay, 40 mg of sample was digested with 1 mg of Collagenase Type IA (from Clostridium histolyticum; ≥260 CDU/mg solid; CDU = Collagen digestion units; Sigma-Aldrich, MO, USA) overnight. 100 ml of standard/ sample was added to 200µl of the DMMB reagent solution (40 mM NaCl, 40 mM glycine, 46 μM DMMB, pH 3.0) and read for absorbance at 540 nm. The amount of sulphated GAGs present was estimated by comparing the samples with chondroitin sulphate as standard.

**Section 1.2 Sample preparation for composite material characterization by FT-IR and XPS:**

The presence of an adhered polymer layer on the decellularized BP was assessed by physical peeling of the polymer from the decellularized tissue as well as by viewing the Bio-Hybrid samples under a stereo microscope (Trinocular Stereo Boom Microscope, Amscope). The interaction between the decellularized BP and the PCL-Ch blend was assessed and confirmed by a) SEM, b) fourier-transform infrared spectroscopy (FT-IR) and x-ray photon spectroscopy (XPS). a) SEM: The surface morphology of the electrospun PCL-Ch and Bio-Hybrid scaffolds were observed by SEM. The samples were fixed with 2.5% glutaraldehyde overnight and serially dehydrated up to 100% ethanol followed by air drying for imaging. The samples were sputter coated in vacuum with an electrically conductive 5 nm thick layer of Gold/Palladium alloy using a precision etching coating system (Denton desktop). Images were then recorded with a high-resolution SEM (Topcon DS 130f). Cross sectional images of the Bio-Hybrid scaffolds were also analyzed for confirming the interaction of polymer with the decellularized tissue. b) FT-IR spectra was recorded for PCL, Ch, PCL-Ch blend, decellularized BP and Bio-Hybrid samples to identify the differences in their functional groups. The samples were air dried, and spectra were taken in the wavelength region 400 cm-1 to 4000 cm-1. C) XPS: near surface elemental and chemical composition of the PCL-Ch blend, decellularized BP and Bio-Hybrid samples were recorded using Thermo K-alpha XPS. The samples were air dried, and finally washed in iso-propyl ethanol. The surface to be analyzed was carefully cut with laboratory scissors without touching the surface and the analysis was done in vacuum. The major elemental composition was recorded, and detailed carbon scanning was done to understand the chemical bonds present in the different groups tested. The binding energy in eV versus the counts for each element was analyzed to understand the differences between the materials.

**Section 1.3 Assessment of peel strength of polymer from decellularized core and the shear stress in a continuous flow loop with different degrees of constriction in the composite:**

**Peel strength of polymer from decellularized core in the composite scaffold**: The strength required to peel the polymer was quantified (n=4) using a customized setup where the polymer was peeled at an angle of 180°. In our setup, Bio-Hybrid samples were cut and taped to a flat surface. Polymer was peeled at the edge and grasped using a clip that was attached to a pulley and a weight hanger using silk thread. The silk was tied to the clip in such a way that the thread lies in the center of the clip such that force is equally distributed across the polymer. Slotted weights were added in increments to the weight hanger to peel the polymer. A ruler was taped next to the sample to measure the peel distance. The addition of the weights was noted, and the corresponding displacement of the polymer was visualized and recorded using a camera that was mounted vertically. The peeling of the polymer was plotted as load vs distance peeled. The peel strength was calculated as Load/ area (width * length).

**Effect of shear stress on Bio-Hybrid scaffolds in a continuous flow loop:** Decellularized BP and Bio-Hybrid samples were subjected to shear stress that is experienced by normal blood vessels for 24 hours to understand the changes that occur to the material surface due to shear stress. A continuous flow loop was set up with a roller pump and a reservoir to exert a shear stress of 15 dynes/cm^2^ that was calculated using the formula $t_{wall}=\frac{32mQ}{pD^{3}}$ where $m$ = viscosity, Q =flow and D=diameter (4). Viscosity was considered equivalent to blood {glycerin: water (40:60), 0.035 poise} with a laminar steady flow, and diameter to be equivalent to a large artery (4). Samples were sutured in the form of 0.25-inch conduits and subjected to continuous flow for 24 hours. In another experiment, a portion of the samples were constricted to about 50% to mimic the natural narrowing of the blood vessels *in vivo* and to study shear. After 24 hours, the lumen of the samples were photographed and analyzed using SEM to investigate the effect of shear of the luminal surface.

**Section 1.4** **Constitutive Modeling:**

Following a similar theoretical framework in Sacks 2000, the pericardial tissue was modeled as an incompressible, homogeneous, and hyperelastic material with a strain energy function *W* using an exponential Fung Model form of *W* and neglecting shear components of the strain tensor*:*

$W(Q)=\frac{c}{2}\left( e^{Q}-1 \right)\mathrm{EQ}$ (Eq. 1)

$$Q(E_{ij})= \left( A_{1}E_{11}^{2}+A_{2}E_{22}^{2}+2A_{3}E_{11}E_{22} \right)$$

where *c* and *A_i_* are material constants and *E* is the Green-Lagrange strain. The modeled in-plane 2^nd^ Piola-Kirchoff stress ***S_ij_*** components are derived from the two-dimensional strain energy function *W*:

$\boldsymbol{S}_{\boldsymbol{ij}}=\frac{\partial W}{\partial E_{ij}}$ (Eq. 2)

Individual data sets of 2^nd^ Piola-Kirchoff and Green-Lagrange were fit to Eq. 1 by minimizing the objective function:

$e= \sum_{j=1}^{n} \left[ \left( \sigma_{11}^{\mathrm{mod}}-\sigma_{11}^{exp} \right)_{j}^{2}+ \left( \sigma_{22}^{\mathrm{mod}}-\sigma_{22}^{exp} \right)_{j}^{2} \right]$ (Eq. 3)

with fmincon optimizing function in MATLAB, where superscripts *mod* and *exp* refer to model predicted and experimental values of stress with the following constraints (2):

$$c>0, A_{1}> \left| A_{3} \right|, \mathrm{and} A_{2}> \left| A_{3} \right|$$

To evaluate the degree of material anisotropy, an anisotropy index was calculated based on the fitted material constants as follows (3):

$Anisotropy=\min\left[ \frac{A_{1}+A_{3}}{A_{2}+A_{3}}, \frac{A_{2}+A_{3}}{A_{1}+A_{3}} \right]$ (Eq. 4)

Where a value of 1 indicates a perfectly isotropic material response and values closer to 0 suggest the material is highly anisotropic.

**Section 1.5. *In-vitro* valve interstitial cell isolation for biocompatibility studies:**

**Cell isolation:** Porcine valve interstitial cells were isolated for the biocompatibility studies using an established protocol described previously (5).The mitral valve was excised from a porcine heart acquired from a local abattoir. The valve was washed with sterile PBS and incubated with 2mg/mL collagenase type II for 5 min in DMEM media. The endothelium was scraped using a sterile blade and the valve tissue was further homogenized and digested in collagenase for another 1 hr. The tissue homogenate was filtered using a 70 μm nylon mesh and then centrifuged at 2000 rpm for 5 min. The pellet with interstitial cells was plated in DMEM and 2X antibiotics. Biocompatibility was tested using cell adhesion studies, where valve interstitial cells were seeded on the Biohybrid and decellularized BP samples, cultured for 48 hr, and then were stained with rhodamine phalloidin and counter stained with DAPI. The cell seeded scaffolds were observed under microscope (Axioscope A1, Carl Zeiss Microscopy, LLC) and photographs were taken to observe the cell adhesion on the material.

**Supplementary Figure Legends:**

**Supplemental Figure 1:** Decellularized and Bio-Hybrid samples subjected to a shear of 30 dynes/cm^2^. The figure shows photographs of the two pericardia after subjecting to flow and the electron microscopy images of the control (no flow), decellularized BP and the Bio-Hybrid samples.

**Supplemental Figure 2:** Uniaxial upper tangent modulus (UTM) and lower tangent modulus (LTM) of untreated BP, decellularized BP and Bio-Hybrid samples in kPa, n=7. Data is represented as mean + standard deviation.

**Supplemental Figure 3:** Individual biaxial stress-strain curves in the circumferential and longitudinal directions for each loading condition, in the untreated BP (left column), decellularized BP (middle), and Bio-Hybrid groups (right column). Individual samples are represented in different colors.

**Supplement References:**

1. Jahnavi S, Kumary TV, Bhuvaneshwar GS, Natarajan TS, Verma RS. Engineering of a polymer layered bio-hybrid heart valve scaffold. *Mater Sci Eng C Mater Biol Appl* (2015) 51:263–273. doi:10.1016/j.msec.2015.03.009

2. Sun W, Sacks MS. Finite element implementation of a generalized Fung-elastic constitutive model for planar soft tissues. *Biomechanics and Modeling in Mechanobiology* (2005) 4:190–199. doi:10.1007/s10237-005-0075-x

3. Rassoli A, Fatouraee N, Guidoin R, Zhang Z. Comparison of tensile properties of xenopericardium from three animal species and finite element analysis for bioprosthetic heart valve tissue. *Artificial Organs* (2020) 44:278–287. doi:10.1111/aor.13552

4. Ku DN. BLOOD FLOW IN ARTERIES. (1996)37.

5. Pathological Remodeling of Mitral Valve Leaflets from Unphysiologic Leaflet Mechanics after Undersized Mitral Annuloplasty to Repair Ischemic Mitral Regurgitation | Journal of the American Heart Association. Available at: https://www.ahajournals.org/doi/10.1161/JAHA.118.009777 [Accessed December 10, 2019]
