# Supplementary figures and images for: A Biohybrid Material With Extracellular Matrix Core and Polymeric Coating as a Cell Honing Cardiovascular Tissue Substitute
Source: Front Cardiovasc Med. 2022 Mar 24;9:807255. doi: 10.3389/fcvm.2022.807255 (PMC8987446; doi:10.3389/fcvm.2022.807255)

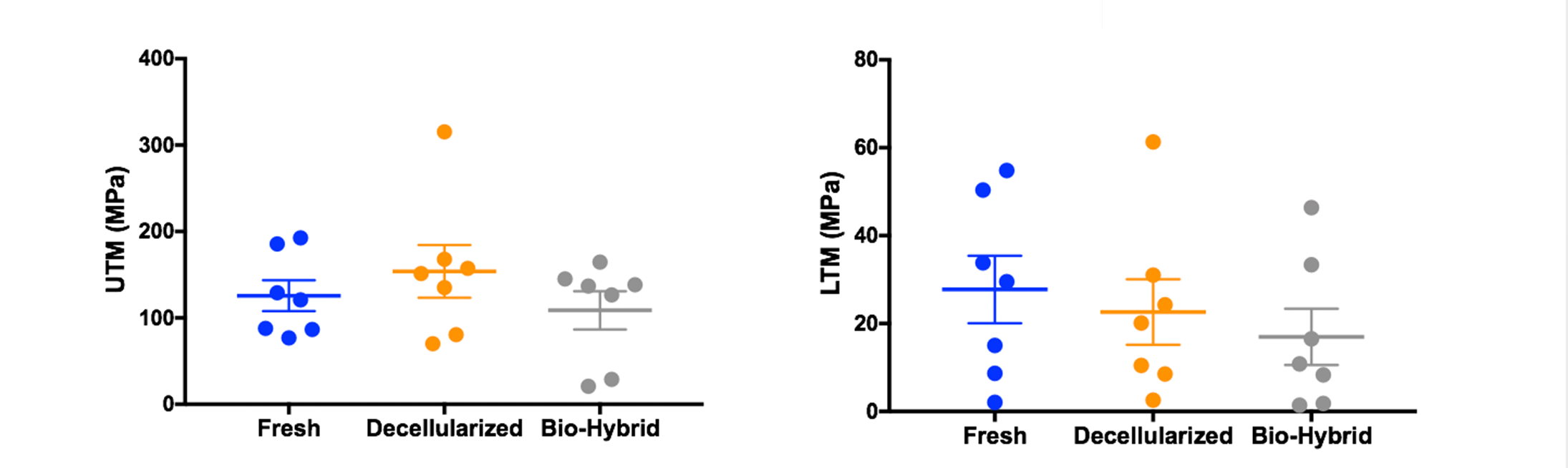

Supplement: Supplementary file 3 [file Image_2.TIF]
